# Finite-time *H*_∞_ synchronization control for coronary artery chaos system with input and state time-varying delays

**DOI:** 10.1371/journal.pone.0266706

**Published:** 2022-04-08

**Authors:** Charuwat Chantawat, Thongchai Botmart

**Affiliations:** Department of Mathematics, Faculty of Science, Khon Kaen University, Khon Kaen, Thailand; Lanzhou University of Technology, CHINA

## Abstract

This is the first time for studying the issue of finite-time *H*_∞_ synchronization control for the coronary artery chaos system (CACS) with input and state time-varying delays. Feedback control is planned for finite-time of synchronization CACS. By constructing the Lyapunov-Krasovskii functional (LKF) is derived for finite-time stability criteria of CACS with interval and continuous differential time-varying delays. We use Wirtinger-based integral inequality to evaluate the upper bound of the time derivative of the LKF. We apply the single integral form and the double integral form of the integral inequality, according to Wirtinger-based integral inequality, to ensure that the feedback controller for synchronization has good performance with disturbance and time-varying delay. The new sufficient finite-time stability conditions have appeared in the form of linear matrix inequalities (LMIs). Numerical checks can be performed using the LMI toolbox in MATLAB. A numerical example is presented to demonstrate the success of the proposed methods. This resultant is less conservative than the resultants available in the previous works.

## 1 Introduction

In recent decades, the synchronization of chaotic systems get attention a lot of attention in many areas such as biomedical, electronics, finance, economics, neural network, and so on [1–5]. In particular, CACS synchronization is an important field. CACS plays a vital role in our lives as it provides enough oxygen and sustenance to the heart throughout the day. Therefore, the integrity of the system is critical. Several effective methods are used to achieve the synchronization between the healthy CACS and diseased CACS such as *H*_∞_ control [6–9], mixed *H*_∞_ and passive performance index [10], adaptive control [11, 12], fuzzy control [13], observer-based control [14, 15] and state-feedback control [16]. Particularly, Zhang et al. [6] studied problems of the synchronization CACS with input disturbances and input time-delay depending on *H*_∞_ control. Li et al. [7] investigated *H*_∞_ control for CACS via free-matrix-based integral inequality with time-delay. The authors in [8] studied uncertain CACS of synchronization controller design depending on Wirtinger integral inequality with input saturation and time delay. The authors in [9] considered the CACS for *H*_∞_ synchronization problems with input time-varying delay and input disturbances. Harshavarthini et al. [10] considered the finite-time synchronization of the CACS system with mixed *H*_∞_ and passive performance index. Li [11] studied the CACS with the adaptive controller depending on the backstepping method to approve local and global boundedness of the system. Wang et al. [13] studied the fuzzy state feedback controller for fuzzy-model-based CACS with state time-delay. Zhao et al. [14] investigated the observer-based *H*_∞_ control for synchronization CACS with time-delay under the external and state uncertainty. On the contrary, the time delay in treatment can have severe consequences for human life and lead to death. Furthermore, the time delay in drug consumption and medicine absorption also degrades system performance and can significantly increase the risk of human life. Therefore, the delay in treatment plays a key role. In addition, Wu et al. [16] investigated CACS for state-feedback synchronization control with interval time-varying delay. Especially in the CACS, it is necessary to predict and diagnose a blockage in the myocardium within a specified period of time to ensure human life is safe. Therefore, a rapid perception of the control system’s work is required. In particular, certain emergency drugs should be consumed at a specific time to reduce the decomposition of oxygen to the myocardium.

In many systems, consideration of the long-time behavior of status variables is not enough because the state variable values during the temporary period may be too large or unrealistic before reaching the equilibrium point. In a chemical process, for instance, the temperature inside a container must be maintained within certain criteria for a period of time for the chemicals to take effect. This situation has been commonly known as finite-time stability (FTS) introduced by Dorato in 1961 [17]. As a result, many researchers are more interested in studying the FTS of various systems. Many researchers have presented criteria that guarantee FTS of various systems with finding the smallest upper bound of the norm square of state variables or finding the maximum time that guarantees values of the state variables to be within the given bounds for a certain time. Some examples of FTS of linear systems with constant delay are shown in [18–25]. [26–30] study on linear systems with time-varying delays for FTS, FTS for synchronization neural networks [31–34] and FTS on other systems [35–41].

In CACS, in particular, it is necessary to predict and diagnose myocardial function within a given time to save our lives. Therefore, quick perception of the efficiency of a control system is desired, and in especially, certain emergency drug intakes should be taken at precise times to reduce the deterioration of the oxygen delivered to the heart muscle. Despite its advantages, finite-time analysis has been one of the most influential and indispensable tools in stabilizing many real-world problems.

As mentioned above, FTS is one of the critical topics that should have been further studied. Thus, in this research, we investigate the finite-time synchronization of CACS with input time-varying delay. In addition, the main contributions of this work are listed as follows;

- This is the first time for studying the finite-time *H*_∞_ synchronization control for CACS containing the input and state time-varying delay is defined. Remarkably, we take the state time-varying delays, which are not considered in [6, 9–11, 14–16].
- A novel LKF is derived for the finite-time *H*_∞_ synchronization controller for CACS with input and state time-varying delay.
- Improve criteria of guaranteeing FTS of CACS with input and state time-varying delay.

In this article, we divide the remainder into four sections. In Section 2, we introduce the CACS and review important definitions and lemmas. A new synchronization criterion for finite-time synchronization of CACS with input and state time-varying delays is shown in Section 3. A numerical simulation is given in Section 4 to show the simulation results of the trajectories of the healthy and diseased CACS. The conclusion is shown in Section 5.

## 2 Problem statement and preliminaries

This document uses the following notation: $R^{q}$ denotes the *q*-dimensional space; $R^{q\times r}$ represents real value matrix with dimension *q* × *r*; *I* represents the identity matrix with appropriate dimensions; *P*^*T*^ refers to the transpose of matrix *P*; *P* is symmetric if *P* = *P*^*T*^; λ(*P*) represents all the eigenvalue of *P*; λ_max_(*P*) = max{*Re* λ: λ ∈ λ(*P*)}; λ_min_(*P*) = min{*Re* λ: λ ∈ λ(*P*)}; *P* < 0 or *P* > 0 represents that the matrix *P* is a symmetric and negative or positive definite matrix; If *P*, *Q* are symmetric, *P* > *Q* interprets as *P* − *Q* is the positive definite matrix. The symmetric terms in the matrix are represented by *. The following norm is used: ∥⋅∥ supersedes the Euclidean vector norm and diag{…} represents a block diagonal matrix and $col\left\{a_{1},a_{2},...,a_{n}\right\}={\left[a_{1}^{T},a_{2}^{T},...,a_{n}^{T}\right]}^{T}$.

The CACS mathematical model is described as follows:

$$\begin{array}{ccc} \dot{r_{1}} & = & -\beta r_{1}-cr_{2},\\ \dot{r_{2}} & = & -\left(\sigma +\beta \sigma \right)r_{1}-\left(\sigma +c\sigma \right)r_{2}+\sigma r_{1}^{3}+E\text{cos}\varpi t, \end{array}$$ (1)

where *r*_1_ is the change of the radius of the blood vessel, *r*_2_ represents the pressure change of the blood vessel, *E* cos *ϖt* represents the periodical stimulating disturbance term, *β*, *c* and *σ* are the system parameters.

The finite-time synchronization of CACS with input and state time-varying delay. Based on (1), the healthy CACS with the state time-varying delays is written as follows:

$$\begin{array}{ccc} \dot{\chi }\left(t\right) & = & A\chi \left(t\right)+\hat{A}\chi \left(t-\eta \left(t\right)\right)+Cf\left(\chi \left(t\right)\right)+\hat{C}g\left(\chi \left(t-\eta \left(t\right)\right)\right)+G\left(t\right). \end{array}$$ (2)

The diseased CACS with the input and state time-varying delays is written as follows:

$$\begin{array}{ccc} \dot{\upsilon }\left(t\right) & = & A\upsilon \left(t\right)+\hat{A}\upsilon \left(t-\eta \left(t\right)\right)+Cf\left(\upsilon \left(t\right)\right)+\hat{C}g\left(\upsilon \left(t-\eta \left(t\right)\right)\right)+G\left(t\right)\\ & & +D\varpi (t)+u(t-\eta (t)), \end{array}$$ (3)

where *A*, $\hat{A}$, *C*, $\hat{C}$, and *D* are the real constant matrices determined by the value of *β*, *c*, *σ* and *E*, $f\left(\chi \left(t\right)\right)=\left[0,\chi_{1}^{3}\left(t\right)\right]$, $g\left(\chi \left(t-\eta \left(t\right)\right)\right)=\left[0,\chi_{1}^{3}\left(t-\eta \left(t\right)\right)\right]$, *G*(*t*) = [0, 0.3 cos *ϖt*], *χ*(*t*) = [*χ*_1_(*t*), *χ*_2_(*t*)]^*T*^, *υ*(*t*) = [*υ*_1_(*t*), *υ*_2_(*t*)]^*T*^ are the state vectors of the healthy and diseased CACS respectively. *ϖ*(*t*) = [*ϖ*_1_(*t*), *ϖ*_2_(*t*)]^*T*^ is the disturbance vectors. *u*(*t* − *η*(*t*)) is control input vector. The continuous input and state time-varying delay functions satisfy:

$$\begin{array}{c} 0\leq \eta_{1}\leq \eta \left(t\right)\leq \eta_{2},\;\dot{\eta }\left(t\right)\leq \rho , \end{array}$$ (4)

where *η*_1_, *η*_2_, *ρ* are known real constant scalars and we denote *η*_12_ = *η*_2_ − *η*_1_, *η*_1*t*_ = *η*(*t*) − *η*_1_, *η*_2*t*_ = *η*_2_ − *η*(*t*).

**Remark 1** *CACS delay is caused by a series of blood transport and biochemical reactions. Therefore, we will call it a state delay. Input delay is often caused by drug absorption or other factors during treatment. This is a complex process. In actual treatment, things that will affect the time it takes for the drug to be absorbed are the patient’s gender, age, and personal status. For the convenience of the study, we suppose input delay and state delay are the same*.

Given *ϵ*(*t*) = *υ*(*t*) − *χ*(*t*), we can get the error system by (2) and (3):

$$\begin{array}{ccc} \dot{\epsilon }\left(t\right) & = & A\epsilon \left(t\right)+\hat{A}\epsilon \left(t-\eta \left(t\right)\right)+Cf\left(\epsilon \left(t\right)\right)+\hat{C}g\left(\epsilon \left(t-\eta \left(t\right)\right)\right)\\ & & +D\varpi (t)+u(t-\eta (t)), \end{array}$$ (5)

where *f*(*ϵ*(*t*)) = *f*(*υ*(*t*)) − *f*(*χ*(*t*)), *g*(*ϵ*(*t* − *η*(*t*))) = *g*(*υ*(*t* − *η*(*t*))) − *g*(*χ*(*t* − *η*(*t*))). We want to synchronize diseased CACS (3) with healthy CACS (2) through the appropriate *u*(*t* − *η*(*t*)) taking into account the delay in drug administration and drug absorption. We can design a time-varying input delay feedback controller as follows:

$$\begin{array}{ccc} u(t-\eta (t)) & = & \tilde{K}\epsilon \left(t-\eta \left(t\right)\right), \end{array}$$ (6)

where $\tilde{K}$ is the gain matrix of control input. By compiling (5) and (6), the error system becomes

$$\begin{array}{ccc} \dot{\epsilon }\left(t\right) & = & A\epsilon \left(t\right)+\hat{A}\epsilon \left(t-\eta \left(t\right)\right)+Cf\left(\epsilon \left(t\right)\right)+\hat{C}g\left(\epsilon \left(t-\eta \left(t\right)\right)\right)+D\varpi \left(t\right)\\ & & +\tilde{K}\epsilon \left(t-\eta \left(t\right)\right). \end{array}$$ (7)

**Remark 2** *This is the first time for studying the finite-time synchronization of CACS* (5) *contains the input and state time-varying delay is defined. If* $\hat{C}=0$ *the error system* (5) *turns into the error system considered by* [6] *and if* $\hat{A}=0$ *and* $\hat{C}=0$ *the error system* (5) *turns into the error system considered by* [9–11, 14–16]. *We can see that the finite-time CACS synchronization of the previous works is already included in our task. This can be considered a special case of finite-time CACS synchronization*.

**Assumption 1** *The function f(χ(t), υ(t), t) and g(χ(t − η(t)), υ(t − η(t)), t)) satisfy*

$$\begin{array}{ccc} ∥f(\upsilon (t))-f(\chi (t))∥ & \leq & ∥L_{f}\left(\upsilon \left(t\right)-\chi \left(t\right)\right)∥,\\ ∥g(\upsilon (t-\eta (t)))-g(\chi (t-\eta (t)))∥ & \leq & ∥L_{g}\left(\upsilon \left(t-\eta \left(t\right)\right)-\chi \left(t-\eta \left(t\right)\right)\right)∥, \end{array}$$

*where L_f_ and L_g_ mean the Lipschitz constant matrix*.

**Definition 1** [28] *Given a matrix U > 0 and three positive real constants* *ς*_1_, *ς*_2_, *T*_*f*_ *with* *ς*_1_ < *ς*_2_, *the time-delay system described by* (7) *and delay condition as in* (4) *is said to be finite-time stable with respect to* (*ς*_1_, *ς*_2_, *T*_*f*_, *η*_2_), *if* ${\text{sup}}_{-\eta_{2}\leq s\leq 0}\left\{\epsilon^{T}\left(s\right)U\epsilon \left(s\right),{\dot{\epsilon }}^{T}\left(s\right)U\dot{\epsilon }\left(s\right)\right\}\leq \varsigma_{1}$ *then* *ϵ*^*T*^(*t*)*Uϵ*(*t*) < *ς*_2_, ∀*t* ∈ [0, *T*_*f*_].

**Definition 2** [8] *Under zero initial conditions, the error system* (7) *is based on the H*_∞_ *performance index*.

$$\begin{array}{c} \int_{0}^{T_{f}}\epsilon^{T}\left(s\right)\epsilon \left(s\right)ds\leq \gamma^{2}\int_{0}^{T_{f}}w^{T}\left(s\right)w\left(s\right)ds, \end{array}$$

*where T*_*f*_ > 0 *represents a sufficiently sizeable real constant, γ* > 0 *is the disturbance attenuation rate*.

**Lemma 1** [9] *Given a matrix Z* > 0, *for derivative functions* $\omega \in \left[\tau_{1},\tau_{2}\right]\to R^{n}$, *we obtain*

$$\begin{array}{c} \int_{\tau_{1}}^{\tau_{2}}{\dot{\omega }}^{T}\left(s\right)Z\dot{\omega }\left(s\right)ds\geq \frac{1}{\tau_{12}}{\left[\begin{array}{c} \sigma_{1}\\ \sigma_{2} \end{array}\right]}^{T}[\begin{array}{cc} Z & 0\\ {}* & Z \end{array}][\begin{array}{c} \sigma_{1}\\ \sigma_{2} \end{array}], \end{array}$$

*where*

$$\begin{array}{ccc} \sigma_{1} & = & \omega \left(\tau_{1}\right)-\omega \left(\tau_{2}\right),\\ \sigma_{2} & = & \sqrt{3}\omega \left(\tau_{1}\right)+\sqrt{3}\omega \left(\tau_{2}\right)-\frac{2\sqrt{3}}{\tau_{2}-\tau_{1}}\int_{\tau_{1}}^{\tau_{2}}\omega \left(s\right)ds. \end{array}$$

**Lemma 2** [42] *For a matrix Z* > 0, *scalars μ and ν with μ* < *ν and a continuous differential function* $\omega :\left[\mu ,\nu \right]\to R^{n}$, *the following integral inequalities are considered*:

$$\begin{array}{ccc} \int_{\mu }^{\nu }\int_{u}^{\nu }{\dot{\omega }}^{T}\left(s\right)Z\dot{\omega }\left(s\right)dsdu & \geq & 2\Omega_{1}^{T}Z\Omega_{1}+4\Omega_{2}^{T}Z\Omega_{2}+6\Omega_{3}^{T}Z\Omega_{3}, \end{array}$$ (8)

*where*

$$\begin{array}{ccc} \kappa & = & \nu -\mu ,\\ \Omega_{1} & = & \omega \left(\nu \right)-\frac{1}{\kappa }\int_{\mu }^{\nu }\omega \left(s\right)ds,\\ \Omega_{2} & = & \omega \left(\nu \right)+\frac{2}{\kappa }\int_{\mu }^{\nu }\omega \left(s\right)ds-\frac{6}{\kappa^{2}}\int_{\mu }^{\nu }\int_{u}^{\nu }\omega \left(s\right)dsdu,\\ \Omega_{3} & = & \omega \left(\nu \right)-\frac{3}{\kappa }\int_{\mu }^{\nu }\omega \left(s\right)ds+\frac{24}{\kappa^{2}}\int_{\mu }^{\nu }\int_{u}^{\nu }\omega \left(s\right)dsdu-\frac{60}{\kappa^{3}}\int_{\mu }^{\nu }\int_{u}^{\nu }\int_{r}^{\nu }\omega \left(s\right)dsdrdu. \end{array}$$

**Remark 3** *In Assumption 1, We suppose that the nonlinear functions f(χ(t), υ(t), t) and g(χ(t − η(t)), υ(t − η(t)), t)) satisfy Lipschitz’s condition. In solving LMIs, the Lipschitz constant is used for limiting nonlinear conditions. L_f_ and L_g_ refer to the Lipschitz constant matrix*.

## 3 Main results

Before introducing the main result, the following notations are defined for simplicity

$$\begin{array}{ccc} e_{i} & = & [0_{2\times (i-1)2}\;\;I\;\;0_{2\times (15-i)2}],i=1,2,...,15,\\ \xi (t) & = & col\{\epsilon \left(t\right),\epsilon \left(t-\eta_{1}\right),\epsilon \left(t-\eta_{2}\right),\epsilon \left(t-\eta \left(t\right)\right),\frac{1}{\eta_{1}}\int_{t-\eta_{1}}^{t}\epsilon \left(s\right)ds,\frac{1}{\eta_{2}}\int_{t-\eta_{2}}^{t}\epsilon \left(s\right)ds,\\ & & \frac{1}{\eta_{1t}}\int_{t-\eta (t)}^{t-\eta_{1}}\epsilon \left(s\right)ds,\frac{1}{\eta_{2t}}\int_{t-\eta_{2}}^{t-\eta (t)}\epsilon \left(s\right)ds,\frac{1}{\eta_{1}^{2}}\int_{t-\eta_{1}}^{t}\int_{r}^{t}\epsilon \left(s\right)dsdr,\\ & & \frac{1}{\eta_{2}^{2}}\int_{t-\eta_{2}}^{t}\int_{r}^{t}\epsilon \left(s\right)dsdr,\frac{1}{\eta_{1}^{3}}\int_{t-\eta_{1}}^{t}\int_{u}^{t}\int_{r}^{t}\epsilon \left(s\right)dsdrdu,\\ & & \frac{1}{\eta_{2}^{3}}\int_{t-\eta_{2}}^{t}\int_{u}^{t}\int_{r}^{t}\epsilon \left(s\right)dsdrdu,f\left(\epsilon \left(t\right)\right),g\left(\epsilon \left(t-\eta \left(t\right)\right)\right),\varpi \left(t\right)\}. \end{array}$$

Now, we provide a stability criterion for the error system (7) with time-varying delay *η*(*t*) satisfy (4).

**Theorem 1** *Given a matrix U > 0, positive scalars ς*_1_, *ς*_2_, *T*, *η*_1_, *η*_2_, *α and any matrix L_f_, L_g_. The error systems* (7) *satisfying Assumption* 1 *and the condition* (4) *is finite-time stable, if there exist positive scalar λ_km_, (m* = 1, 2, …, 10), *δ*_1_, *δ*_2_, *positive definite matrices P, Q_i_, R*_*j*_, $W_{j}\in R^{n\times n}$, (*i* = 1, 2, 3, 4, *j* = 1, 2) *any matrices S*_1_, *S*_2_ *with proper dimensions such that the following LMIs hold*:

$$\begin{array}{c} {}[\begin{array}{cc} R_{2} & S_{1}\\ {}* & R_{2} \end{array}]\geq 0,\;\;\;[\begin{array}{cc} R_{2} & S_{2}\\ {}* & R_{2} \end{array}]\geq 0, \end{array}$$ (9)

$$\begin{array}{c} \Psi =[\begin{array}{cc} \Psi_{11} & \Psi_{12}\\ {}* & \Psi_{22} \end{array}]<0, \end{array}$$ (10)

$$\begin{array}{ccc} & & \lambda_{k1}I<\tilde{P}<\lambda_{k2}I,{\tilde{Q}}_{1}<\lambda_{k3}I,\;\;{\tilde{Q}}_{2}<\lambda_{k4}I,{\tilde{Q}}_{3}<\lambda_{k5}I,\;\;{\tilde{Q}}_{4}<\lambda_{k6}I,\\ & & {\tilde{R}}_{1}<\lambda_{k7}I,{\tilde{R}}_{2}<\lambda_{k8}I,\;\;{\tilde{W}}_{1}<\lambda_{k9}I,\;\;{\tilde{W}}_{2}<\lambda_{k10}I, \end{array}$$ (11)

$$\begin{array}{ccc} & & e^{\alpha T_{f}}\Lambda \varsigma_{1}-\lambda_{k1}\varsigma_{2}<0, \end{array}$$ (12)

*where*

$$\begin{array}{ccc} \Psi_{11} & = & \vartheta_{11}+\vartheta_{12}+\vartheta_{13}+\vartheta_{14}+\vartheta_{15}+\vartheta_{16}+\vartheta_{17}+\vartheta_{18}-e_{1}^{T}\alpha Pe_{1},\\ \Psi_{12} & = & [\eta_{1}\Xi^{T}R_{1},\eta_{12}\Xi^{T}R_{2},\frac{\eta_{1}}{\sqrt{2}}\Xi^{T}W_{1},\frac{\eta_{2}}{\sqrt{2}}\Xi^{T}W_{2}],\\ \Psi_{22} & = & diag\{-R_{1},-R_{2},-W_{1},-W_{2}\},\\ \Xi & = & Ae_{1}+\hat{A}e_{4}+\tilde{K}e_{4}+Ce_{13}+\hat{C}e_{14}+De_{15},\\ \Gamma_{1} & = & e_{1}-e_{2},\Gamma_{2}=\sqrt{3}e_{1}+\sqrt{3}e_{2}-2\sqrt{3}e_{5},\\ \Gamma_{3} & = & e_{2}-e_{4},\Gamma_{4}=\sqrt{3}e_{2}+\sqrt{3}e_{4}-2\sqrt{3}e_{7},\\ \Gamma_{5} & = & e_{4}-e_{3},\Gamma_{6}=\sqrt{3}e_{4}+\sqrt{3}e_{3}-2\sqrt{3}e_{8},\\ \pi_{1} & = & e_{1}-e_{5},\pi_{2}=e_{1}+2e_{5}-6e_{9},\pi_{3}=e_{1}-3e_{5}+24e_{9}-60e_{11},\\ \pi_{4} & = & e_{1}-e_{6},\pi_{5}=e_{1}+2e_{6}-6e_{10},\pi_{6}=e_{1}-3e_{6}+24e_{10}-60e_{12},\\ \vartheta_{11} & = & e_{1}^{T}\left[P+P^{T}\right]\Xi ,\\ \vartheta_{12} & = & e_{1}^{T}\left(Q_{1}+Q_{2}+Q_{3}\right)e_{1}-\left(1-\rho \right)e_{4}^{T}Q_{1}e_{4}+e_{2}^{T}\left(Q_{4}-Q_{2}\right)e_{2}\\ & & -e_{3}^{T}\left(Q_{3}-Q_{4}\right)e_{3},\\ \vartheta_{13} & = & -\Gamma_{1}^{T}R_{1}\Gamma_{1}-\Gamma_{2}^{T}R_{1}\Gamma_{2},\\ \vartheta_{14} & = & -\Gamma_{3}^{T}R_{2}\Gamma_{3}-\Gamma_{4}^{T}R_{2}\Gamma_{4}-\Gamma_{5}^{T}R_{2}\Gamma_{5}-\Gamma_{6}^{T}R_{2}\Gamma_{6}\\ & & -\Gamma_{3}^{T}S_{1}\Gamma_{5}-\Gamma_{5}^{T}S_{1}^{T}\Gamma_{3}-\Gamma_{4}^{T}S_{2}\Gamma_{6}-\Gamma_{6}^{T}S_{2}^{T}\Gamma_{4},\\ \vartheta_{15} & = & -2\pi_{1}^{T}W_{1}\pi_{1}-4\pi_{2}^{T}W_{1}\pi_{2}-6\pi_{3}^{T}W_{1}\pi_{3},\\ \vartheta_{16} & = & -2\pi_{4}^{T}W_{2}\pi_{4}-4\pi_{5}^{T}W_{2}\pi_{5}-6\pi_{6}^{T}W_{2}\pi_{6},\\ \vartheta_{17} & = & e_{1}^{T}\delta_{1}L_{f}^{T}L_{f}e_{1}-e_{13}^{T}\delta_{1}Ie_{13},\\ \vartheta_{18} & = & +e_{4}^{T}\delta_{2}L_{g}^{T}L_{g}e_{4}-e_{14}^{T}\delta_{2}Ie_{14},\\ \Lambda & = & \lambda_{k2}+\eta_{2}\lambda_{k3}+\eta_{1}\lambda_{k4}+\eta_{2}\lambda_{k5}+\eta_{12}\lambda_{k6}+\frac{\eta_{1}^{3}}{2}\lambda_{k7}+\frac{\eta_{12}^{3}}{2}\lambda_{k8}+\frac{\eta_{1}^{3}}{6}\lambda_{k9}\\ & & +\frac{\eta_{2}^{3}}{6}\lambda_{k10},\\ \lambda_{k1} & = & \lambda_{\text{min}}\left(\tilde{P}\right),\;\;\lambda_{k2}=\lambda_{\text{max}}\left(\tilde{P}\right),\;\;\lambda_{k3}=\lambda_{\text{max}}\left({\tilde{Q}}_{1}\right),\;\;\lambda_{k4}=\lambda_{\text{max}}\left({\tilde{Q}}_{2}\right),\\ \lambda_{k5} & = & \lambda_{\text{max}}\left({\tilde{Q}}_{3}\right),\;\;\lambda_{k6}=\lambda_{\text{max}}\left({\tilde{Q}}_{4}\right),\;\;\lambda_{k7}=\lambda_{\text{max}}\left({\tilde{R}}_{1}\right),\;\;\lambda_{k8}=\lambda_{\text{max}}\left({\tilde{R}}_{2}\right),\\ \lambda_{k9} & = & \lambda_{\text{max}}\left({\tilde{W}}_{1}\right),\;\;\lambda_{k10}=\lambda_{\text{max}}\left({\tilde{W}}_{2}\right). \end{array}$$

*Proof*: Consider the LKF candidate as follows:

$$\begin{array}{c} V\left(\epsilon \left(t\right)\right)=\sum_{i=1}^{4}V_{i}\left(\epsilon \left(t\right)\right), \end{array}$$ (13)

where

$$\begin{array}{ccc} V_{1}\left(\epsilon \left(t\right)\right) & = & \epsilon^{T}\left(t\right)P\epsilon \left(t\right),\\ V_{2}\left(\epsilon \left(t\right)\right) & = & \int_{t-\eta (t)}^{t}\epsilon^{T}\left(s\right)Q_{1}\epsilon \left(s\right)ds+\int_{t-\eta_{1}}^{t}\epsilon^{T}\left(s\right)Q_{2}\epsilon \left(s\right)ds\\ & & +\int_{t-\eta_{2}}^{t}\epsilon^{T}\left(s\right)Q_{3}\epsilon \left(s\right)ds+\int_{t-\eta_{2}}^{t-\eta_{1}}\epsilon^{T}\left(s\right)Q_{4}\epsilon \left(s\right)ds,\\ V_{3}\left(\epsilon \left(t\right)\right) & = & \eta_{1}\int_{-\eta_{1}}^{0}\int_{\theta }^{t}{\dot{\epsilon }}^{T}\left(s\right)R_{1}\dot{\epsilon }\left(s\right)dsd\theta +\eta_{12}\int_{-\eta_{2}}^{-\eta_{1}}\int_{\theta }^{t}{\dot{\epsilon }}^{T}\left(s\right)R_{2}\dot{\epsilon }\left(s\right)dsd\theta ,\\ V_{4}\left(\epsilon \left(t\right)\right) & = & \int_{t-\eta_{1}}^{t}\int_{\theta }^{t}\int_{r}^{t}{\dot{\epsilon }}^{T}\left(s\right)W_{1}\dot{\epsilon }\left(s\right)dsdrd\theta +\int_{t-\eta_{2}}^{t}\int_{\theta }^{t}\int_{r}^{t}{\dot{\epsilon }}^{T}\left(s\right)W_{2}\dot{\epsilon }\left(s\right)dsdrd\theta . \end{array}$$

The time derivative of *V*(*ϵ*(*t*)) can be defined as follows:

$$\begin{array}{ccc} {\dot{V}}_{1}\left(\epsilon \left(t\right)\right) & = & 2\epsilon^{T}\left(t\right)P\dot{\epsilon }\left(t\right)\\ & = & \xi^{T}\left(t\right)\vartheta_{11}\xi \left(t\right), \end{array}$$ (14)

$$\begin{array}{ccc} {\dot{V}}_{2}\left(\epsilon \left(t\right)\right) & \leq & \epsilon^{T}\left(t\right)\left[Q_{1}+Q_{2}+Q_{3}\right]\epsilon \left(t\right)-\left(1-\rho \right)\epsilon^{T}\left(t-\eta \left(t\right)\right)Q_{1}\epsilon \left(t-\eta \left(t\right)\right)\\ & & +\epsilon^{T}\left(t-\eta_{1}\right)\left(Q_{4}-Q_{2}\right)\epsilon \left(t-\eta_{1}\right)-\epsilon^{T}\left(t-\eta_{2}\right)\left(Q_{3}+Q_{4}\right)\epsilon \left(t-\eta_{2}\right)\\ & = & \xi^{T}\left(t\right)\vartheta_{12}\xi \left(t\right), \end{array}$$ (15)

$$\begin{array}{ccc} {\dot{V}}_{3}\left(\epsilon \left(t\right)\right) & = & {\dot{\epsilon }}^{T}\left(t\right)\left[\eta_{1}^{2}R_{1}+\eta_{12}^{2}R_{2}\right]\dot{\epsilon }\left(t\right)-\eta_{1}\int_{t-\eta_{1}}^{t}{\dot{\epsilon }}^{T}\left(s\right)R_{1}\dot{\epsilon }\left(s\right)ds\\ & & -\eta_{12}\int_{t-\eta (t)}^{t-\eta_{1}}{\dot{\epsilon }}^{T}\left(s\right)R_{2}\dot{\epsilon }\left(s\right)ds-\eta_{12}\int_{t-\eta_{2}}^{t-\eta (t)}{\dot{\epsilon }}^{T}\left(s\right)R_{2}\dot{\epsilon }\left(s\right)ds, \end{array}$$ (16)

$$\begin{array}{ccc} {\dot{V}}_{4}\left(\epsilon \left(t\right)\right) & = & {\dot{\epsilon }}^{T}\left(t\right)[\frac{\eta_{1}^{2}}{2}W_{1}+\frac{\eta_{2}^{2}}{2}W_{2}]\dot{\epsilon }\left(t\right)-\int_{t-\eta_{1}}^{t}\int_{r}^{t}{\dot{\epsilon }}^{T}\left(s\right)W_{1}\dot{\epsilon }\left(s\right)dsdr\\ & & -\int_{t-\eta_{2}}^{t}\int_{r}^{t}{\dot{\epsilon }}^{T}\left(s\right)W_{2}\dot{\epsilon }\left(s\right)dsdr. \end{array}$$ (17)

Using Lemma 1, we get

$$\begin{array}{ccc} \zeta_{1}\left(t\right) & \leq & -{\left[\begin{array}{c} \psi_{1}\left(t\right)\\ \psi_{2}\left(t\right) \end{array}\right]}^{T}\left[\begin{array}{cc} R_{1} & 0\\ {}* & R_{1} \end{array}\right]\left[\begin{array}{c} \psi_{1}\left(t\right)\\ \psi_{2}\left(t\right) \end{array}\right]\\ & = & \xi^{T}\left(t\right)\vartheta_{13}\xi \left(t\right), \end{array}$$ (18)

$$\begin{array}{ccc} \zeta_{2}\left(t\right) & \leq & -\frac{1}{\phi_{1}}{\left[\begin{array}{c} \psi_{3}\left(t\right)\\ \psi_{4}\left(t\right) \end{array}\right]}^{T}\left[\begin{array}{cc} R_{2} & 0\\ {}* & R_{2} \end{array}\right]\left[\begin{array}{c} \psi_{3}\left(t\right)\\ \psi_{4}\left(t\right) \end{array}\right], \end{array}$$ (19)

$$\begin{array}{ccc} \zeta_{3}\left(t\right) & \leq & -\frac{1}{\phi_{2}}{\left[\begin{array}{c} \psi_{5}\left(t\right)\\ \psi_{6}\left(t\right) \end{array}\right]}^{T}\left[\begin{array}{cc} R_{2} & 0\\ {}* & R_{2} \end{array}\right]\left[\begin{array}{c} \psi_{5}\left(t\right)\\ \psi_{6}\left(t\right) \end{array}\right], \end{array}$$ (20)

where

$$\begin{array}{ccc} \zeta_{1}\left(t\right) & = & -\eta_{1}\int_{t-\eta_{1}}^{t}{\dot{\epsilon }}^{T}\left(s\right)R_{1}\dot{\epsilon }\left(s\right)ds,\\ \zeta_{2}\left(t\right) & = & -\eta_{12}\int_{t-\eta (t)}^{t-\eta_{1}}{\dot{\epsilon }}^{T}\left(s\right)R_{2}\dot{\epsilon }\left(s\right)ds,\\ \zeta_{3}\left(t\right) & = & -\eta_{12}\int_{t-\eta_{2}}^{t-\eta (t)}{\dot{\epsilon }}^{T}\left(s\right)R_{2}\dot{\epsilon }\left(s\right)ds,\\ \phi_{1} & = & \frac{\eta_{1t}}{\eta_{12}},\;\;\phi_{2}=\frac{\eta_{2t}}{\eta_{12}},\\ \psi_{1}\left(t\right) & = & \epsilon \left(t\right)-\epsilon \left(t-\eta_{1}\right),\\ \psi_{2}\left(t\right) & = & \sqrt{3}\epsilon \left(t\right)+\sqrt{3}\epsilon \left(t-\eta_{1}\right)-\frac{2\sqrt{3}}{\eta_{1}}\int_{t-\eta_{1}}^{t}\epsilon \left(s\right)ds,\\ \psi_{3}\left(t\right) & = & \epsilon \left(t-\eta_{1}\right)-\epsilon \left(t-\eta \left(t\right)\right),\\ \psi_{4}\left(t\right) & = & \sqrt{3}\epsilon \left(t-\eta_{1}\right)+\sqrt{3}\epsilon \left(t-\eta \left(t\right)\right)-\frac{2\sqrt{3}}{\eta_{1t}}\int_{t-\eta (t)}^{t-\eta_{1}}\epsilon \left(s\right)ds,\\ \psi_{5}\left(t\right) & = & \epsilon \left(t-\eta \left(t\right)\right)-\epsilon \left(t-\eta_{2}\right),\\ \psi_{6}\left(t\right) & = & \sqrt{3}\epsilon \left(t-\eta \left(t\right)\right)+\sqrt{3}\epsilon \left(t-\eta_{2}\right)-\frac{2\sqrt{3}}{\eta_{2t}}\int_{t-\eta_{2}}^{t-\eta (t)}\epsilon \left(s\right)ds. \end{array}$$

It is clear that the real numbers *ϕ*_1_ and *ϕ*_2_ correspond to *ϕ*_1_ > 0, *ϕ*_2_ > 0 and *ϕ*_1_ + *ϕ*_2_ = 1, then suggest an appropriate dimensional matrix *S*_1_ and *S*_2_ such that

$$\begin{array}{c} \left[\begin{array}{cc} R_{2} & S_{1}\\ {}* & R_{2} \end{array}\right]\geq 0,\;\;\;\left[\begin{array}{cc} R_{2} & S_{2}\\ {}* & R_{2} \end{array}\right]\geq 0. \end{array}$$ (21)

By reciprocally convex to inequalities (19) and (20), we obtain

$$\begin{array}{ccc} \zeta_{2}\left(t\right)+\zeta_{3}\left(t\right) & \leq & -\frac{1}{\phi_{1}}\psi_{3}^{T}\left(t\right)R_{2}\psi_{3}\left(t\right)-\frac{1}{\phi_{2}}\psi_{5}^{T}\left(t\right)R_{2}\psi_{5}\left(t\right)\\ & & -\frac{1}{\phi_{1}}\psi_{4}^{T}\left(t\right)R_{2}\psi_{4}\left(t\right)-\frac{1}{\phi_{2}}\psi_{6}^{T}\left(t\right)R_{2}\psi_{6}\left(t\right)\\ & \leq & -{\left[\begin{array}{c} \psi_{3}\left(t\right)\\ \psi_{5}\left(t\right) \end{array}\right]}^{T}\left[\begin{array}{cc} R_{2} & S_{1}\\ {}* & R_{2} \end{array}\right]\left[\begin{array}{c} \psi_{3}\left(t\right)\\ \psi_{5}\left(t\right) \end{array}\right]\\ & & -{\left[\begin{array}{c} \psi_{4}\left(t\right)\\ \psi_{6}\left(t\right) \end{array}\right]}^{T}\left[\begin{array}{cc} R_{2} & S_{2}\\ {}* & R_{2} \end{array}\right]\left[\begin{array}{c} \psi_{4}\left(t\right)\\ \psi_{6}\left(t\right) \end{array}\right]\\ & = & \xi^{T}\left(t\right)\vartheta_{14}\xi \left(t\right). \end{array}$$ (22)

Using Lemma 2, we obtain

$$\begin{array}{c} -\int_{t-\eta_{1}}^{t}\int_{r}^{t}{\dot{\epsilon }}^{T}\left(s\right)W_{1}\dot{\epsilon }\left(s\right)dsdr\leq -\xi^{T}\left(t\right)\vartheta_{15}\xi \left(t\right), \end{array}$$ (23)

$$\begin{array}{c} -\int_{t-\eta_{2}}^{t}\int_{r}^{t}{\dot{\epsilon }}^{T}\left(s\right)W_{2}\dot{\epsilon }\left(s\right)dsdr\leq -\xi^{T}\left(t\right)\vartheta_{16}\xi \left(t\right). \end{array}$$ (24)

From the Assumption 1, we have

$$\begin{array}{ccc} 0 & \leq & \delta_{1}\epsilon^{T}\left(t\right)L_{f}^{T}L_{f}\epsilon \left(t\right)-\delta_{1}f^{T}\left(\epsilon \left(t\right)\right)f\left(\epsilon \left(t\right)\right)\\ & = & \xi^{T}\left(t\right)\vartheta_{17}\xi \left(t\right), \end{array}$$ (25)

$$\begin{array}{ccc} 0 & \leq & \delta_{2}\epsilon^{T}\left(t-\eta \left(t\right)\right)L_{g}^{T}L_{g}\epsilon \left(t-\eta \left(t\right)\right)\\ & & -\delta_{2}g^{T}\left(\epsilon \left(t-\eta \left(t\right)\right)\right)g\left(\epsilon \left(t-\eta \left(t\right)\right)\right)\\ & = & \xi^{T}\left(t\right)\vartheta_{18}\xi \left(t\right). \end{array}$$ (26)

Combining (14)–(26), we get

$$\begin{array}{c} \dot{V}\left(\epsilon \left(t\right)\right)\leq \xi^{T}\left(t\right)ϒ\xi \left(t\right)+\alpha V_{1}\left(\epsilon \left(t\right)\right)<\xi^{T}\left(t\right)ϒ\xi \left(t\right)+\alpha V\left(\epsilon \left(t\right)\right), \end{array}$$ (27)

where

$$\begin{array}{ccc} ϒ & = & \Psi_{11}+\Delta ,\\ \Delta & = & \Xi^{T}\left[\eta_{1}^{2}R_{1}+\eta_{12}^{2}R_{2}+\frac{\eta_{1}^{2}}{2}W_{1}+\frac{\eta_{2}^{2}}{2}W_{2}\right]\Xi . \end{array}$$

Applying Schur complement lemma the inequalities ϒ is equivalent to Ψ < 0, from (27) we get

$$\begin{array}{ccc} \dot{V}\left(\epsilon \left(t\right)\right) & < & \alpha V(\epsilon (t)). \end{array}$$ (28)

Multiplying the above inequality by *e*^−*αt*^ and integrating form 0 to *t* with *t* ∈ [0, *T*_*f*_], we have

$$\begin{array}{c} V\left(\epsilon \left(t\right)\right)<e^{\alpha T_{f}}V\left(\epsilon \left(0\right)\right), \end{array}$$ (29)

with

$$\begin{array}{ccc} V(\epsilon (0)) & = & \epsilon^{T}\left(0\right)P\epsilon \left(0\right)+\int_{-\eta (0)}^{0}\epsilon^{T}\left(s\right)Q_{1}\epsilon \left(s\right)ds+\int_{-\eta_{1}}^{0}\epsilon^{T}\left(s\right)Q_{2}\epsilon \left(s\right)ds\\ & & +\int_{-\eta_{2}}^{0}\epsilon^{T}\left(s\right)Q_{3}\epsilon \left(s\right)d+\int_{-\eta_{2}}^{-\eta_{1}}\epsilon^{T}\left(s\right)Q_{4}\epsilon \left(s\right)ds\\ & & +\eta_{1}\int_{-\eta_{1}}^{0}\int_{\theta }^{0}{\dot{\epsilon }}^{T}\left(s\right)R_{1}\dot{\epsilon }\left(s\right)dsd\theta +\eta_{12}\int_{-\eta_{2}}^{-\eta_{1}}\int_{\theta }^{0}{\dot{\epsilon }}^{T}\left(s\right)R_{2}\dot{\epsilon }\left(s\right)dsd\theta \\ & & +\int_{-\eta_{1}}^{0}\int_{\theta }^{0}\int_{r}^{0}{\dot{\epsilon }}^{T}\left(s\right)W_{1}\dot{\epsilon }\left(s\right)dsdrd\theta +\int_{-\eta_{2}}^{0}\int_{\theta }^{0}\int_{r}^{0}{\dot{\epsilon }}^{T}\left(s\right)W_{2}\dot{\epsilon }\left(s\right)dsdrd\theta . \end{array}$$

Since $I=U^{\frac{1}{2}}U^{-\frac{1}{2}}=U^{-\frac{1}{2}}U^{\frac{1}{2}}$, $\tilde{P}=U^{-\frac{1}{2}}PU^{-\frac{1}{2}}$, $\tilde{Q_{i}}=U^{-\frac{1}{2}}Q_{i}U^{-\frac{1}{2}}$, $\tilde{R_{j}}=U^{-\frac{1}{2}}R_{j}U^{-\frac{1}{2}}$, $\tilde{W_{j}}=U^{-\frac{1}{2}}W_{j}U^{-\frac{1}{2}}$, (*i* = 1, 2, 3, 4, *j* = 1, 2), thus *V*(*ϵ*(0)) can be written as

$$\begin{array}{ccc} V(\epsilon (0)) & = & \epsilon^{T}\left(0\right)U^{\frac{1}{2}}\tilde{P}U^{\frac{1}{2}}\epsilon \left(0\right)+\int_{-\eta (0)}^{0}\epsilon^{T}\left(s\right)U^{\frac{1}{2}}\tilde{Q_{1}}U^{\frac{1}{2}}\epsilon \left(s\right)ds\\ & & +\int_{-\eta_{1}}^{0}\epsilon^{T}\left(s\right)U^{\frac{1}{2}}\tilde{Q_{2}}U^{\frac{1}{2}}\epsilon \left(s\right)ds+\int_{-\eta_{2}}^{0}\epsilon^{T}\left(s\right)U^{\frac{1}{2}}\tilde{Q_{3}}U^{\frac{1}{2}}\epsilon \left(s\right)ds\\ & & +\int_{-\eta_{2}}^{-\eta_{1}}\epsilon^{T}\left(s\right)U^{\frac{1}{2}}\tilde{Q_{4}}U^{\frac{1}{2}}\epsilon \left(s\right)ds+\eta_{1}\int_{-\eta_{1}}^{0}\int_{\theta }^{0}{\dot{\epsilon }}^{T}\left(s\right)U^{\frac{1}{2}}\tilde{R_{1}}U^{\frac{1}{2}}\dot{\epsilon }\left(s\right)dsd\theta \\ & & +\eta_{12}\int_{-\eta_{2}}^{-\eta_{1}}\int_{\theta }^{0}{\dot{\epsilon }}^{T}\left(s\right)U^{\frac{1}{2}}\tilde{R_{2}}U^{\frac{1}{2}}\dot{\epsilon }\left(s\right)dsd\theta \\ & & +\int_{-\eta_{1}}^{0}\int_{\theta }^{0}\int_{r}^{0}{\dot{\epsilon }}^{T}\left(s\right)U^{\frac{1}{2}}\tilde{W_{1}}U^{\frac{1}{2}}\dot{\epsilon }\left(s\right)dsdrd\theta \\ & & +\int_{-\eta_{2}}^{0}\int_{\theta }^{0}\int_{r}^{0}{\dot{\epsilon }}^{T}\left(s\right)U^{\frac{1}{2}}\tilde{W_{2}}U^{\frac{1}{2}}\dot{\epsilon }\left(s\right)dsdrd\theta \\ & \leq & [\lambda_{\text{max}}\left(\tilde{P}\right)+\eta_{2}\lambda_{\text{max}}\left(\tilde{Q_{1}}\right)+h_{1}\lambda_{\text{max}}\left(\tilde{Q_{2}}\right)+\eta_{2}\lambda_{\text{max}}\left(\tilde{Q_{3}}\right)+\eta_{12}\lambda_{\text{max}}\left(\tilde{Q_{4}}\right)\\ & & +\frac{\eta_{1}^{3}}{2}\lambda_{\text{max}}\left(\tilde{R_{1}}\right)+\frac{\eta_{12}^{3}}{2}\lambda_{\text{max}}\left(\tilde{R_{2}}\right)+\frac{\eta_{1}^{3}}{6}\lambda_{\text{max}}\left(\tilde{W_{1}}\right)+\frac{\eta_{2}^{3}}{6}\lambda_{\text{max}}\left(\tilde{W_{2}}\right)]\\ & & \underset{-\eta_{2}\leq s\leq 0}{\text{sup}}\left\{\epsilon^{T}\left(s\right)U\epsilon \left(s\right),{\dot{\epsilon }}^{T}\left(s\right)U\dot{\epsilon }\left(s\right)\right\}\\ & \leq & [\lambda_{\text{max}}\left(\tilde{P}\right)+\eta_{2}\lambda_{\text{max}}\left(\tilde{Q_{1}}\right)+\eta_{1}\lambda_{\text{max}}\left(\tilde{Q_{2}}\right)+\eta_{2}\lambda_{\text{max}}\left(\tilde{Q_{3}}\right)+\eta_{12}\lambda_{\text{max}}\left(\tilde{Q_{4}}\right)\\ & & +\frac{\eta_{1}^{3}}{2}\lambda_{\text{max}}\left(\tilde{R_{1}}\right)+\frac{\eta_{12}^{3}}{2}\lambda_{\text{max}}\left(\tilde{R_{2}}\right)+\frac{\eta_{1}^{3}}{6}\lambda_{\text{max}}\left(\tilde{W_{1}}\right)+\frac{\eta_{2}^{3}}{6}\lambda_{\text{max}}\left(\tilde{W_{2}}\right)]\varsigma_{1}\\ & = & \Lambda \varsigma_{1}. \end{array}$$

Because $V\left(\epsilon \left(t\right)\right)\geq V_{1}\left(\epsilon \left(t\right)\right)=\epsilon^{T}\left(t\right)U^{\frac{1}{2}}\tilde{P}U^{\frac{1}{2}}\epsilon \left(t\right)\geq \lambda_{\text{min}}\left(\tilde{P}\right)\epsilon^{T}\left(t\right)U\epsilon \left(t\right)$. Thus, for any *t* ∈ [0, *T*_*f*_], we obtain

$$\begin{array}{c} \epsilon^{T}\left(t\right)U\epsilon \left(t\right)<\frac{e^{\alpha T_{f}}\Lambda \varsigma_{1}}{\lambda_{k1}}<\varsigma_{2}. \end{array}$$ (30)

Hence, the condition (12) holds and the proof is complete.

**Remark 4** *The condition defined in the Theorem 1 can be used for analyzing the stability of error systems based on unknown* $\tilde{K}$. *For the sake of solving the problem of the matrix* $\tilde{K}$, *sufficient conditions can be provided as follows*:

**Theorem 2** *Given a matrix U* > 0, *positive scalars ς*_1_, *ς*_2_, *T*, *η*_1_, *η*_2_, *α and any matrix L_f_, L_g_. If there exist positive scalar θ_m_, (m* = 1, 2, …, 10), ${\bar{\delta }}_{1}$, ${\bar{\delta }}_{2}$, *positive definite matrices X*, ${\bar{Q}}_{i}$, ${\bar{R}}_{j}$, ${\bar{W}}_{j}\in R^{n\times n},\left(i=1,2,3,4,j=1,2\right)$ *any matrix* $Y,{\bar{S}}_{1},{\bar{S}}_{2}$ *with suitable dimensions such that the following LMIs hold*:

$$\begin{array}{c} \left[\begin{array}{cc} {\bar{R}}_{2} & {\bar{S}}_{1}\\ {}* & {\bar{R}}_{2} \end{array}\right]\geq 0,\;\;\;\left[\begin{array}{cc} {\bar{R}}_{2} & {\bar{S}}_{2}\\ {}* & {\bar{R}}_{2} \end{array}\right]\geq 0, \end{array}$$ (31)

$$\begin{array}{c} \bar{\Psi }=\left[\begin{array}{cccc} {\bar{\Psi }}_{11} & {\bar{\Psi }}_{12} & e_{1}XL_{f}^{T} & e_{4}XL_{g}^{T}\\ {}* & -{\bar{\Psi }}_{22} & 0 & 0\\ {}* & * & -\bar{\delta_{1}}I & 0\\ {}* & * & * & -\bar{\delta_{2}}I \end{array}\right]<0, \end{array}$$ (32)

$$\begin{array}{ccc} & & M_{k1}<\Pi_{11}<M_{k2},\;\;\Pi_{12}<M_{k3},\;\;\Pi_{13}<M_{k4},\;\;\Pi_{14}<M_{k5},\\ & & \Pi_{15}<M_{k6},\;\;\Pi_{16}<M_{k7},\;\;\Pi_{17}<M_{k8},\;\;\Pi_{18}<M_{k9},\;\;\Pi_{19}<M_{k10}, \end{array}$$ (33)

$$\begin{array}{ccc} & & e^{\alpha T}\Theta \varsigma_{1}-\theta_{1}\varsigma_{2}<0, \end{array}$$ (34)

*where*

$$\begin{array}{ccc} {\bar{\Psi }}_{11} & = & {\bar{\vartheta }}_{11}+{\bar{\vartheta }}_{12}+{\bar{\vartheta }}_{13}+{\bar{\vartheta }}_{14}+{\bar{\vartheta }}_{15}+{\bar{\vartheta }}_{16}+{\bar{\vartheta }}_{17}-e_{1}^{T}\alpha Xe_{1}\\ & & +e_{1}^{T}Ie_{1}-\gamma^{2}e_{15}^{T}Ie_{15},\\ {\bar{\Psi }}_{12} & = & \left[\eta_{1}{\bar{\Xi }}^{T},\eta_{12}{\bar{\Xi }}^{T},\frac{\eta_{1}}{\sqrt{2}}{\bar{\Xi }}^{T},\frac{\eta_{2}}{\sqrt{2}}{\bar{\Xi }}^{T}\right],\\ {\bar{\Psi }}_{22} & = & diag\{X{\bar{R}}_{1}^{-1}X,X{\bar{R}}_{2}^{-1}X,X{\bar{W}}_{1}^{-1}X,X{\bar{W}}_{2}^{-1}X\},\\ \bar{\Xi } & = & AXe_{1}+\hat{A}Xe_{4}+Ye_{4}+Ce_{13}+\hat{C}e_{14}+De_{15},\\ {\bar{\vartheta }}_{11} & = & e_{1}^{T}\bar{\Xi }+{\bar{\Xi }}^{T}e_{1},\\ \;{\bar{\vartheta }}_{12} & = & e_{1}^{T}\left({\bar{Q}}_{1}+{\bar{Q}}_{2}+{\bar{Q}}_{3}\right)e_{1}-\left(1-\rho \right)e_{4}^{T}{\bar{Q}}_{1}e_{4}+e_{2}^{T}\left({\bar{Q}}_{4}-{\bar{Q}}_{2}\right)e_{2}\\ & & -e_{3}^{T}\left({\bar{Q}}_{3}-{\bar{Q}}_{4}\right)e_{3},\\ {\bar{\vartheta }}_{13} & = & -\Gamma_{1}^{T}{\bar{R}}_{1}\Gamma_{1}-\Gamma_{2}^{T}{\bar{R}}_{1}\Gamma_{2},\\ {\bar{\vartheta }}_{14} & = & -\Gamma_{3}^{T}{\bar{R}}_{2}\Gamma_{3}-\Gamma_{4}^{T}{\bar{R}}_{2}\Gamma_{4}-\Gamma_{5}^{T}{\bar{R}}_{2}\Gamma_{5}-\Gamma_{6}^{T}{\bar{R}}_{2}\Gamma_{6}\\ & & -\Gamma_{3}^{T}{\bar{S}}_{1}\Gamma_{5}-\Gamma_{5}^{T}{\bar{S}}_{1}^{T}\Gamma_{3}-\Gamma_{4}^{T}{\bar{S}}_{2}\Gamma_{6}-\Gamma_{6}^{T}{\bar{S}}_{2}^{T}\Gamma_{4},\\ {\bar{\vartheta }}_{15} & = & -2\pi_{1}^{T}{\bar{W}}_{1}\pi_{1}-4\pi_{2}^{T}{\bar{W}}_{1}\pi_{2}-6\pi_{3}^{T}{\bar{W}}_{1}\pi_{3},\\ {\bar{\vartheta }}_{16} & = & -2\pi_{4}^{T}{\bar{W}}_{2}\pi_{4}-4\pi_{5}^{T}{\bar{W}}_{2}\pi_{5}-6\pi_{6}^{T}{\bar{W}}_{2}\pi_{6},\\ {\bar{\vartheta }}_{17} & = & -e_{13}^{T}{\bar{\delta }}_{1}Ie_{13}-e_{14}^{T}{\bar{\delta }}_{2}Ie_{14},\\ \Theta & = & \theta_{2}+\eta_{2}\theta_{3}+\eta_{1}\theta_{4}+\eta_{2}\theta_{5}+\eta_{12}\theta_{6}+\frac{\eta_{1}^{3}}{2}\theta_{7}+\frac{\eta_{12}^{3}}{2}\theta_{8}+\frac{\eta_{1}^{3}}{6}\theta_{9}+\frac{\eta_{2}^{3}}{6}\theta_{10},\\ \theta_{1} & = & \lambda_{\text{min}}\left(O_{1}\right),\theta_{2}=\lambda_{\text{max}}\left(O_{1}\right),\theta_{3}=\lambda_{\text{max}}\left(O_{2}\right),\theta_{4}=\lambda_{\text{max}}\left(O_{3}\right),\\ \theta_{5} & = & \lambda_{\text{max}}\left(O_{4}\right),\theta_{6}=\lambda_{\text{max}}\left(O_{5}\right),\theta_{7}=\lambda_{\text{max}}\left(O_{6}\right),\theta_{8}=\lambda_{\text{max}}\left(O_{7}\right),\\ \theta_{9} & = & \lambda_{\text{max}}\left(O_{8}\right),\theta_{10}\lambda_{\text{max}}\left(O_{9}\right), \end{array}$$

*then, the error systems* (7) *satisfying Assumption 1 and the condition* (4) *is finite-time stable. In this case, the desired controllers are given as follows*:

$$\begin{array}{ccc} \tilde{K} & = & YX^{-1}. \end{array}$$ (35)

*Proof*: The *H*_∞_ performance will be proved in this theorem. The proof of this theorem is a consequence of Theorem 1. Now by following the Theorem 1 along with the same LKF candidate (13) for any non-zero disturbance *ϖ*(*t*), it is easy to get

$$\begin{array}{c} \dot{V}\left(\epsilon \left(t\right)\right)+\epsilon {\left(t\right)}^{T}\epsilon \left(t\right)-\gamma^{2}\varpi {\left(t\right)}^{T}\varpi \left(t\right)\leq \xi {\left(t\right)}^{T}\left[\Psi_{11}+F\right]\xi \left(t\right), \end{array}$$ (36)

where the elements in Ψ_11_ are same as in (10), $F=e_{1}^{T}Ie_{1}-\gamma^{2}e_{15}^{T}Ie_{15}$.

Now, by using Schur complement lemma and setting *X* = *P*^−1^, then pre-multiplying and post-multiplying with *diag*{*X*, *X*},

$$\begin{array}{c} diag\{\overset{12}{\overset{︷}{X,\cdots ,X}},I,I,I,R_{1}^{-1},R_{2}^{-1},W_{1}^{-1},W_{2}^{-1}\}, \end{array}$$

to (9) and (10) respectively, the inequality in (11) and (12) multiplies by *X* from both left and right sides. By setting

$$\begin{array}{ccc} Y & = & KX,{\bar{Q}}_{i}=XQ_{i}X,{\bar{R}}_{j}=XR_{j}X,{\bar{W}}_{j}=XW_{j}X,{\bar{\delta }}_{j}=\delta_{j}^{-1},\\ & & (i=1,2,3,4,j=1,2),\\ M_{k1} & = & X\left(\lambda_{k1}I\right)X,M_{k2}=X\left(\lambda_{k2}I\right)X,M_{k3}=X\left(\lambda_{k3}I\right)X,M_{k4}=X\left(\lambda_{k4}I\right)X,\\ M_{k5} & = & X\left(\lambda_{k5}I\right)X,M_{k6}=X\left(\lambda_{k6}I\right)X,M_{k7}=X\left(\lambda_{k7}I\right)X,M_{k8}=X\left(\lambda_{k8}I\right)X,\\ M_{k9} & = & X\left(\lambda_{k9}I\right)X,M_{k10}=X\left(\lambda_{k10}I\right)X,\\ \Pi_{11} & = & X\tilde{P}X,\Pi_{12}=X{\tilde{Q}}_{1}X,\Pi_{13}=X{\tilde{Q}}_{2}X,\Pi_{14}=X{\tilde{Q}}_{3}X,\Pi_{15}=X{\tilde{Q}}_{4}X,\\ \Pi_{16} & = & X{\tilde{R}}_{1}X,\Pi_{17}=X{\tilde{R}}_{2}X,\Pi_{18}=X{\tilde{W}}_{1}X,\Pi_{19}=X{\tilde{W}}_{2}X, \end{array}$$

the inequalities (31)–(34) can be attained, which completes the proof.

**Remark 5** *Because Theorem 2 contains nonlinear terms* $X{\bar{R}}_{j}^{-1}X,$ $X{\bar{W}}_{j}^{-1}X,\left(j=1,2\right)$, *the feasible solutions to this problem can be found by the cone complementary linearization algorithm (CCLA). Hence, the inequality* (32) *can be modified using the iterative algorithm*.

*Firstly, we define new variables U*_*j*_ *and Z*_*j*_(*j* = 1, 2), *such that*

$$\begin{array}{ccc} X{\bar{R}}_{j}^{-1}X & \geq & U_{j},\\ X{\bar{W}}_{j}^{-1}X & \geq & Z_{j}, \end{array}$$ (37)

*which can be transformed to*

$$\begin{array}{ccc} \left[\begin{array}{cc} -{\bar{R}}_{j}^{-1} & X^{-1}\\ {}* & -U_{j}^{-1} \end{array}\right] & \leq & 0,\\ \left[\begin{array}{cc} -{\bar{W}}_{j}^{-1} & X^{-1}\\ {}* & -Z_{j}^{-1} \end{array}\right] & \leq & 0,(j=1,2). \end{array}$$ (38)

*By introducing variables X*^−1^ = *X*_*n*_, ${\bar{R}}_{j}^{-1}=J_{j}$, ${\bar{U}}_{j}^{-1}=H_{j}$, ${\bar{W}}_{j}^{-1}=L_{j}$, ${\bar{Z}}_{j}^{-1}=T_{j},\left(j=1,2\right)$, *which is equivalent to*

$$\begin{array}{ccc} \left[\begin{array}{cc} -J_{j} & X_{n}\\ {}* & -H_{j} \end{array}\right] & \leq & 0,\\ \left[\begin{array}{cc} -L_{j} & X_{n}\\ {}* & -T_{j} \end{array}\right] & \leq & 0,(j=1,2). \end{array}$$ (39)

*According to the CCLA, the original problem of Theorem 2 can be replaced by the following minimization problem*.

*Minimize*

$$\begin{array}{c} tr(X_{n}X+\sum_{j=1}^{2}(J_{j}{\bar{R}}_{j}+H_{j}U_{j}+L_{j}{\bar{W}}_{j}+T_{j}{\bar{Z}}_{j})), \end{array}$$

*subject to* (31)–(34), *and*

$$\begin{array}{ccc} \left[\begin{array}{cccc} {\bar{\Psi }}_{11} & {\bar{\Psi }}_{12} & e_{1}XL_{f}^{T} & e_{4}XL_{g}^{T}\\ {}* & O_{22} & 0 & 0\\ {}* & * & -\bar{\delta_{1}}I & 0\\ {}* & * & * & -\bar{\delta_{2}}I \end{array}\right] & < & 0, \end{array}$$ (40)

$$\begin{array}{ccc} \left[\begin{array}{cc} X_{n} & I\\ {}* & X \end{array}\right] & \geq & 0,\left[\begin{array}{cc} J_{j} & I\\ {}* & R_{j} \end{array}\right]\geq 0,\\ \left[\begin{array}{cc} H_{j} & I\\ {}* & U_{j} \end{array}\right] & \geq & 0,\left[\begin{array}{cc} L_{j} & I\\ {}* & W_{j} \end{array}\right]\geq 0,\\ \left[\begin{array}{cc} T_{j} & I\\ {}* & Z_{j} \end{array}\right] & \geq & 0,(j=1,2), \end{array}$$ (41)

*where O*_22_ = *diag*{−*U*_1_, −*U*_2_, −*Z*_1_, −*Z*_2_}.

## 4 Numerical simulation

A numerical simulation is performed to show the performance of the schemes proposed in this section.

The parameters of the healthy CACS (2) and diseased CACS (3) are as follows;

$$\begin{array}{ccc} & & A=\left[\begin{array}{cc} -0.1 & 1.5\\ 0.55 & -0.25 \end{array}\right],\;\;\hat{A}=\left[\begin{array}{cc} -0.05 & 0.2\\ 0.025 & -0.1 \end{array}\right],\;\;C=\left[\begin{array}{cc} 0 & 0\\ 0 & -0.4 \end{array}\right],\\ & & \hat{C}=\left[\begin{array}{cc} 0 & 0\\ 0 & -0.1 \end{array}\right],\;\;D=\left[\begin{array}{cc} 0.2 & 0\\ 0 & 0.1 \end{array}\right],\;\;L_{f}=\left[\begin{array}{cc} 0 & 0\\ 0 & 4.8 \end{array}\right],\\ & & L_{g}=\left[\begin{array}{cc} 0 & 0\\ 0 & 1.2 \end{array}\right],\;\;\chi \left(0\right)=\left[\begin{array}{c} 1\\ 0.5 \end{array}\right],\;\;\upsilon \left(0\right)=\left[\begin{array}{c} -0.5\\ -1 \end{array}\right]. \end{array}$$

For simulation purpose, we assume *ς*_1_ = 0.1, *ς*_2_ = 5, *T*_*f*_ = 10, *α* = 0.1, *ρ* = 0.2, *U* = *I*.

Case 1. When *η*(*t*) = 0.24 + 0.025sin(*t*), *η*_1_ = 0.1, *η*_2_ = 0.55, with disturbance *ϖ*(*t*) = [0.3 sin(40*t*), 0.1 sin(30*t*)]^*T*^.
Case 2. When *η*(*t*) = 0.4 + 0.02sin(*t*), *η*_1_ = 0.1, *η*_2_ = 0.5, with disturbance *ϖ*(*t*) = [0.3 sin(40*t*), 0.1 sin(30*t*)]^*T*^.
Case 3. When *η*(*t*) = 0.3 + 0.05sin(*t*), *η*_1_ = 0.1, *η*_2_ = 0.45, with disturbance *ϖ*(*t*) = [0.09 sin(4*t*), 0.03 sin(5*t*)]^*T*^.
Case 4. When *η*(*t*) = 0.15 + 0.04sin(4*t*), *η*_1_ = 0.1, *η*_2_ = 0.4, with disturbance *ϖ*(*t*) = [0.09 sin(4*t*), 0.03 sin(5*t*)]^*T*^.

For the case 1–4, we obtain an appropriate gain matrix $\tilde{K}$ by solving LMIs (31)–(34) obtained in Theorem 2 and represented in Table 1.

**Table 1:** Gain matrix $\tilde{K}$ for distinct delay and disturbance function.

Case	*η*(*t*)	*ϖ*(*t*)	Gain matrix $\tilde{K}$
1	0.24 + 0.025 sin(*t*)	$\left[\begin{array}{c} 0.3\text{sin}(40t)\\ 0.1\text{sin}(30t) \end{array}\right]$	$\left[\begin{array}{cc} -1.1984 & -0.1004\\ -0.1003 & -0.8445 \end{array}\right]$
2	0.4 + 0.02 sin(*t*)	$\left[\begin{array}{c} 0.3\text{sin}(40t)\\ 0.1\text{sin}(30t) \end{array}\right]$	$\left[\begin{array}{cc} -1.8887 & -0.0806\\ -0.0805 & -1.4075 \end{array}\right]$
3	0.3 + 0.05 sin(*t*)	$\left[\begin{array}{c} 0.09\text{sin}(4t)\\ 0.03\text{sin}(5t) \end{array}\right]$	$\left[\begin{array}{cc} -2.7084 & -0.0748\\ -0.0747 & -2.2705 \end{array}\right]$
4	0.15 + 0.04 sin(4*t*)	$\left[\begin{array}{c} 0.09\text{sin}(4t)\\ 0.03\text{sin}(5t) \end{array}\right]$	$\left[\begin{array}{cc} -3.7204 & -0.0424\\ -0.0423 & -2.8897 \end{array}\right]$

**Fig 1:**
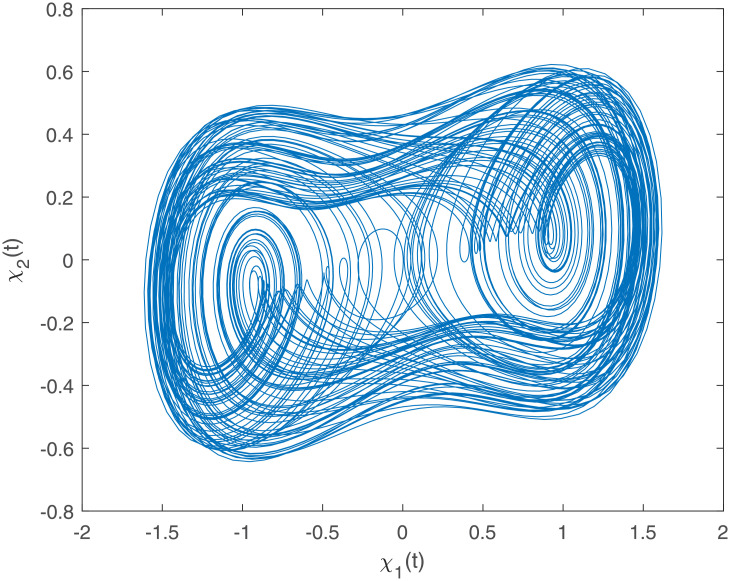
The healthy CACS phase portraits under *ϖ*(*t*) = 0 with no control input.

**Fig 2:**
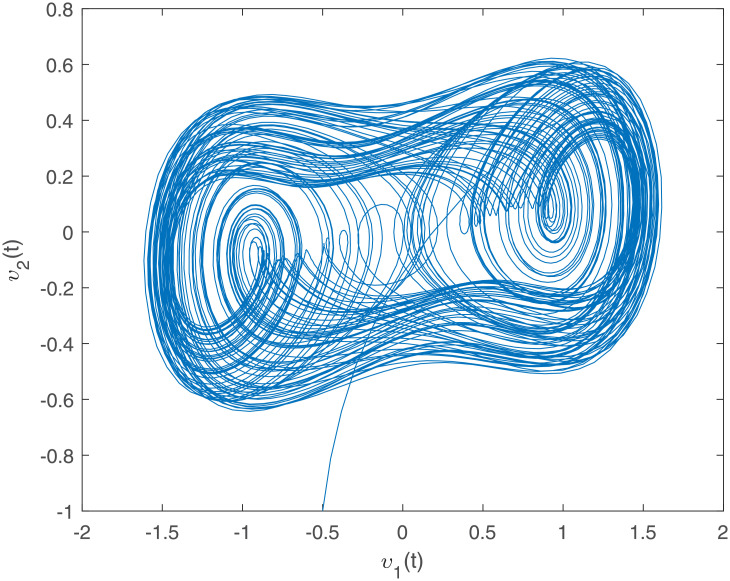
The diseased CACS phase portraits under *ϖ*(*t*) = 0 with no control input.

**Fig 3:**
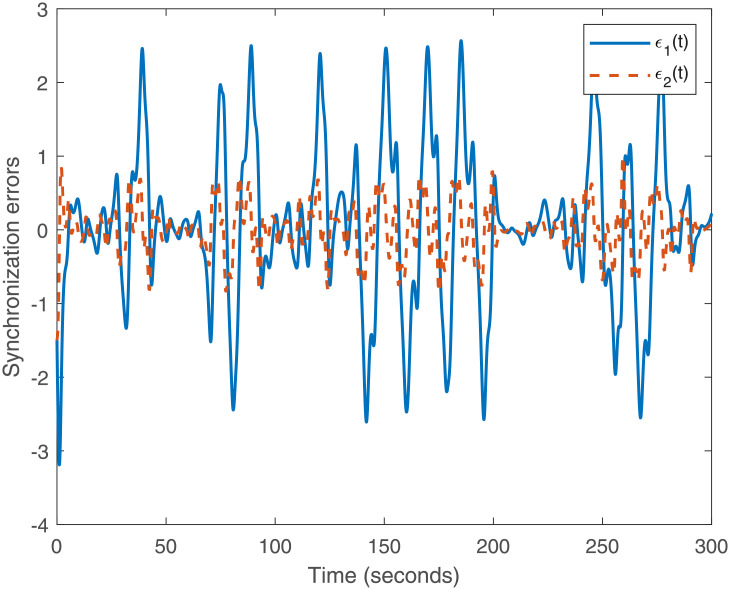
System behavior error under *ϖ*(*t*) = 0 with no control input.

**Fig 4:**
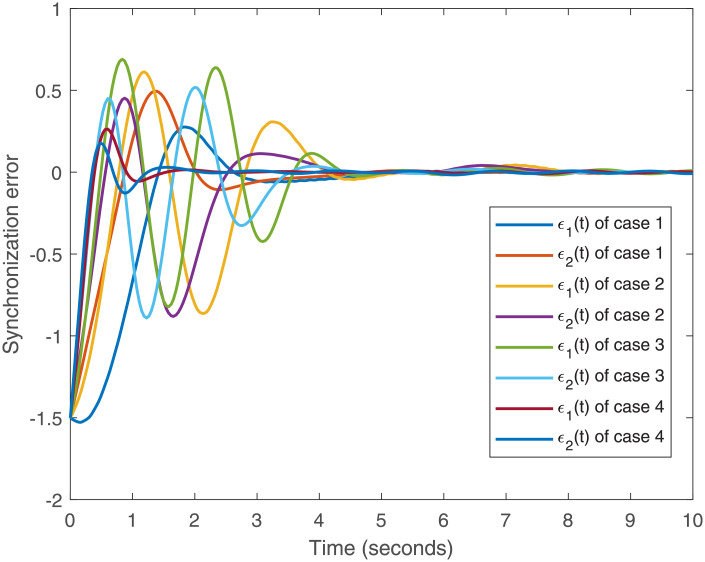
System behavior error under *ϖ*(*t*) ≠ 0 with the control input for the cases 1–4.

**Fig 5:**
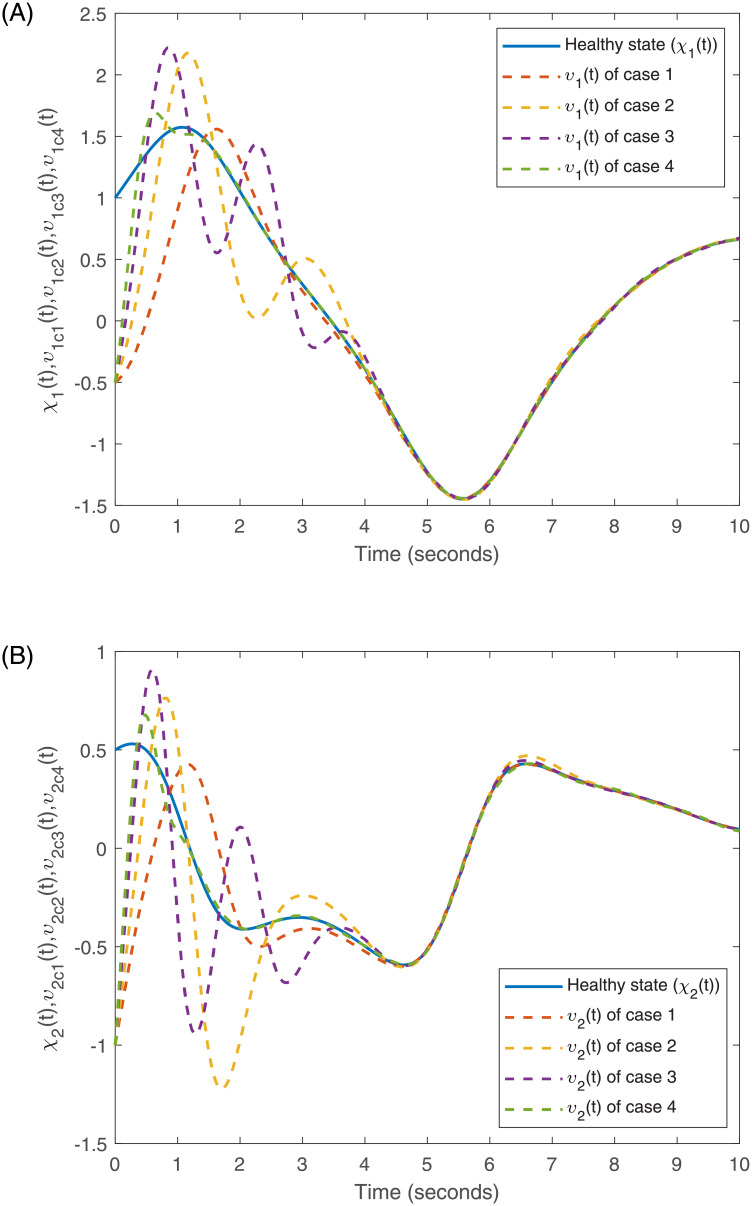
Response of the state for the healthy and diseased CACS for cases 1–4.

**Fig 6:**
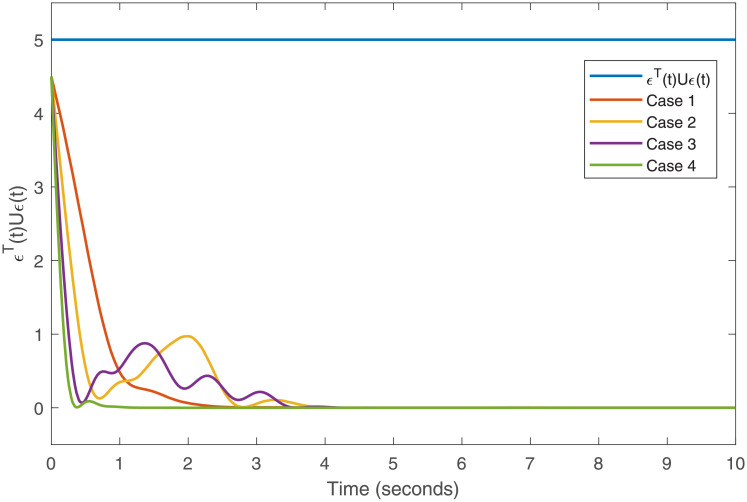
Evaluation of *ϵ*^*T*^(*t*)*Uϵ*(*t*) for the cases 1–4.

**Fig 7:**
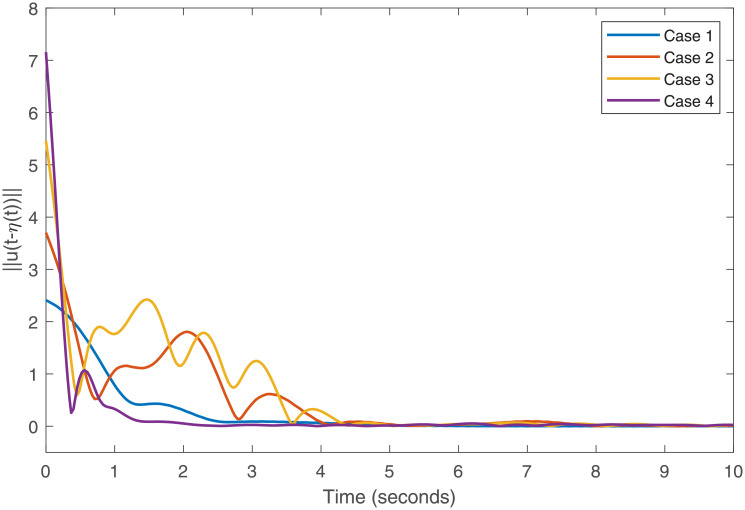
Control response for the cases 1–4.

Accurately, Figs 1–7 show the simulation results associated with the controller designed in (6). Especially, Fig 1 demonstrates the phases of healthy CACS (2) under *ϖ*(*t*) = 0 with no control input. Fig 2 demonstrates the phase of the diseased CACS (3) under *ϖ*(*t*) = 0 with no control input. The error systems between the healthy CACS and diseased CACS without the controller is plotted in Fig 3. Therefore, Fig 3 presents the importance of the regulator in maintaining a normal heart rhythm. Moreover, the synchronization error systems between (2) and (3) through the controller (6) under the various time-vary delays and disturbances for case 1–4 is plotted in Fig 4. The controller, therefore, changes with the upper bound of the time-varying delay with increasing time. In natural treatment, things that will affect the time it takes for the drug to be absorbed are the patient’s gender, age, and personal status. We must endorse the effectiveness of treatment in other cases. The efficiency of our strategy can be expressed as Fig 5. From Fig 5, it is seen that within a short time, the control input can effectively synchronize the diseased system with the health system for the different delay and disturbance input which is shown in Table 1. Fig 6 displays the immediate cognizance of the system (2) and (3) within a specific time period guaranteed by planning the trajectories of *ϵ*^*T*^(*t*)*Uϵ*(*t*) with the finite-time bound *ς*_2_. The control response for all four cases is shown in Fig 7. The designed controller performs a vital role in the synchronization and necessity of today’s studies.

Therefore, from the results of these simulations, it is seen that the proposed controller (6) approves synchronization between healthy and diseased CACS at precise intervals. Significantly, the inherent potential of the developed theoretical results is realized with the minimum attenuation index. In addition, the control vector is resistant to delays and therapeutic risks and maintains the health of diseased CACS even under unpredictable factors.

**Remark 6** *The advantage of this numerical simulation is the lower bound of the delay η*_1_ ≠ 0. *Moreover, we still study the CACS with input and state time-varying delays. Hence, the stability conditions derived in* [6, 9–11, 14–16] *cannot be applied to this simulation*.

## 5 Conclusion

This is the first time studying the finite-time *H*_∞_ synchronization control for CACS containing the input and state time-varying delay is defined. Significantly, the reliable controller is devised to suppress abnormal heart rhythms, which is necessary to supply the heart with nutrients and oxygen all day. This compares to the unpredictable side, for example, drug consumption, emotional volatility, and so on. By constructing a new LKF and using Wirtinger-based inequality, improved single/double integral inequalities and stability criteria conditions are in the term of LMIs, which are sufficient to ensure that the diseased system synchronizes with the health system for a limited time. The simulations show that our synchronization strategy effectively synchronizes the convulsive coronary system with the healthy cardiovascular system under input delay and disturbance. In future work, the results and methods in this work are expected to use to other various systems in real-word application, for instant, *H*_∞_ control [6–9], mixed *H*_∞_ and passive performance index [10], adaptive control [11, 12], fuzzy control [13], observer-based control [14, 15] and state-feedback control [16], projective synchronization of chaotic systems [43] and stochastic differential equations [44]. Furthermore, input delay and state delay will be considered in different values to get more closer to reality.

## Supporting information

S1 File (M)
